# Hybridization Directionality Governs the Interaction Strength between MoS_2_ and Metals

**DOI:** 10.1021/acs.nanolett.5c03200

**Published:** 2025-08-19

**Authors:** Michaela Hanušová, Luka Pirker, Martin Vondráček, Václav Valeš, Casey K. Cheung, Noel Natera Cordero, Amy Carl, Viktor Zólyomi, János Koltai, Ilias Sotiriou, Jens Zscharschuch, Artur Erbe, Roman Gorbachev, Jan Honolka, Otakar Frank, Matěj Velický

**Affiliations:** † J. Heyrovský Institute of Physical Chemistry, 48311 Czech Academy of Sciences (CAS), Dolejškova 2155/3, 182 23 Prague 8, Czech Republic; ‡ Faculty of Chemical Engineering, University of Chemistry and Technology, Prague, Technická 5, 166 28 Prague 6, Czech Republic; ¶ 86889 Institute of Physics, CAS, Na Slovance 1999/2, 182 21 Prague 8, Czech Republic; § Department of Physics and Astronomy, 5292 University of Manchester, Oxford Road, Manchester M13 9PL, United Kingdom; ∥ Hartree Centre, 15585 STFC Daresbury Laboratory, Daresbury WA4 4AD, United Kingdom; ⊥ Department of Biological Physics, 54616 Eötvös Loránd University, Pázmány Péter sétány 1/A, Budapest 1117, Hungary; # Institute of Electronic Packaging Technology, 9169 Technische Universität Dresden, 01062 Dresden, Germany; @ 28414 Institute of Ion Beam Physics and Materials Research, Helmholtz-Zentrum Dresden-Rossendorf, Bautzner Landstrasse 400, 01328 Dresden, Germany; △ 9169 Technische Universität Dresden, 01069 Dresden, Germany

**Keywords:** MoS_2_, metals, electronic structure, hybridization, photoemission spectroscopy, Raman spectroscopy

## Abstract

Gold-assisted exfoliation has emerged as an effective method for producing large-area monolayers of two-dimensional materials, yet its underlying mechanism remains poorly understood. While other metals also hold promise for facilitating large-area exfoliation, their practical application is hindered by oxidation in air. To address this, we fabricate heterostructures of monolayer MoS_2_ with polycrystalline gold, silver, copper, palladium, cobalt, and nickel via direct mechanical exfoliation of bulk molybdenite under controlled atmospheric conditions. Our photoemission spectroscopy, vibrational spectroscopy, and density functional theory results reveal the metal-dependent modification of monolayer MoS_2_. We identify the hybridization directionality and, in particular, the asymmetry between the bottom and top sulfur atoms as previously overlooked key factors in weakening the MoS_2_–MoS_2_ van der Waals interaction, ultimately enabling selective monolayer exfoliation.

## Graphical abstract

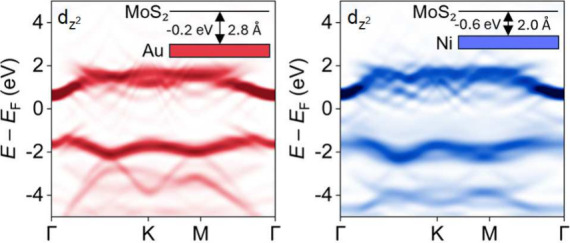

The exploration of the interface between two-dimensional (2D) materials and metals, facilitated by their strong interactions, has revealed the modulation of the band structure by moiré potential,^1^ enabled the observation of charge density waves,^2^ and led to advances in contact engineering.^3^ The recently discovered metal-assisted exfoliation technique broadened the accessibility of the 2D/metal systems to a large research community.^4^ This wave of research was kick-started by several studies in which selective exfoliation of large-area MoS_2_ monolayers on gold was achieved.^5–8^ Subsequent rapid progress in sample fabrication and the resulting discoveries sparked a surge of research efforts in this direction.

The mechanism behind monolayer-selective, gold-assisted exfoliation of transition metal dichalcogenides (TMDCs) has been attributed to the interaction energy,^7,9^ strain,^10–12^ or an interplay of strain and interfacial electrostatics.^13^ In practice, it also depends on the MoS_2_ crystallinity, surface roughness, nobility of the metal, interfacial cleanliness, and contact pressure.^7,11,14–17^ Most studies use gold as the metallic substrate for exfoliation due to its uncomplicated sample preparation, which is underpinned by the remarkable ability of gold to resist surface oxidation. The gold surface suffers from the buildup of organic contamination in air, but its slow onset permits large-area monolayer exfoliation when done within minutes.^7^ Other metals additionally deteriorate due to oxidation, which weakens their adhesion with the 2D material and suppresses exfoliation.^14^ The strong interaction with gold appears to be universal for various 2D materials, including TMDCs, metal halides, and metal thiophosphates.^8^

Exfoliation of 2D materials on non-Au metals had remained elusive until the recent breakthroughs in utilizing the controlled atmosphere of a glovebox or ultrahigh vacuum (UHV).^15,18–20^ A majority of these efforts focus on the four most common TMDCs, i.e., MoS_2_, WS_2_, MoSe_2_, and WSe_2_, due to their environmental stability,^21^ their band gap tunability,^22^ and the experimental evidence of the strong interaction with metals.^19,23^ Deposition of metals on bulk TMDCs has also been successful in preparing large-area monolayers. However, their properties differ from those of the directly exfoliated ones due to the damage to the TMDC lattice induced by the high-energy metal atoms.^3,9,13^

It is currently unclear how the interaction strength and the resulting electronic properties vary for different metals interfacing with 2D materials. To that end, we investigate the interaction between MoS_2_ and six different metals in samples prepared in controlled environments of the glovebox and UHV systems with low concentrations of oxygen, water, and other contaminants. These conditions suppress metal oxidation and facilitate a large-area monolayer MoS_2_ exfoliation expected from the theory.^24^ Photoemission spectroscopy and polarization-dependent Raman spectroscopy, supported by density functional theory (DFT), reveal interrelated changes in the hybridization, vibrational modes, and interaction energies of MoS_2_ that depend on the metallic substrate. Furthermore, the directional hybridization of the MoS_2_ orbitals with the topmost metal orbitals and its asymmetry with respect to the top versus bottom sulfur atoms appear to underpin the metal-assisted exfoliation. The role of various factors in selective monolayer exfoliation has been debated in the literature without a clear consensus, and the effects of hybridization have been largely overlooked.

Our DFT calculations for two different MoS_2_ supercells (details provided in the Supporting Information) reveal a correlation between the interaction energy and equilibrium distance of MoS_2_ and metals (Figure 1a), which reflects the strength of the interaction and corroborates a previous report.^24^ The absolute interaction energies are substantially higher on the metals (from −105 to −832 meV) than the interlayer van der Waals (vdW) energy between MoS_2_ layers (−28 meV). Moreover, there is a 4-fold versus 8-fold difference between the weakest (Au) and strongest (Ni) interaction energies for the large versus small supercells. Nevertheless, our results demonstrate that the interaction energy is not the limiting factor for successful exfoliation as it is more negative than the MoS_2_ interlayer energy for all of these metals. Instead, it has been argued that selective monolayer exfoliation is conditional on the weakening of the interaction between the first and second MoS_2_ layer.^4,25^ However, there has been limited experimental evidence or explanation of what causes the weakening to date.^12^

**Figure 1:**
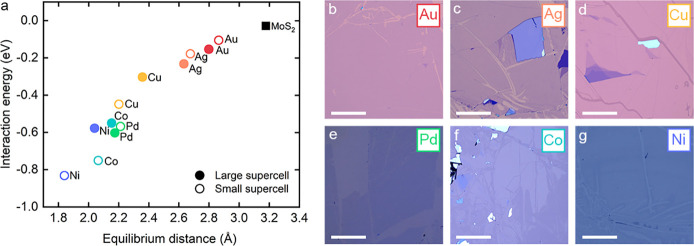
(a) Monolayer MoS_2_–metal interaction energy (per MoS_2_ unit cell) vs equilibrium distance between the top metal layer and the bottom S layer. Two data sets, calculated for larger (filled circles) and smaller (empty circles) supercells with different compensating strain, are shown (further details in Table S1). The black square is the interlayer interaction energy between MoS_2_ layers (per MoS_2_ unit cell) calculated for 3L of MoS_2_. (b–g) Optical images of large-area MoS_2_ monolayers on Au, Ag, Cu, Pd, Co, and Ni, respectively, exfoliated in a glovebox. All scale bars correspond to 200 μm. The optical contrast of the monolayers varies due to the differing optical constants and postexfoliation oxidation of the metals. RGB values were adjusted to achieve maximum contrast between the monolayer and the metal.

Initially, we reproduced previously reported exfoliation of large-area monolayer MoS_2_ by depositing a polycrystalline gold film on a cleaned SiO_2_ substrate and pressing the bulk MoS_2_ crystal against the gold surface immediately after its exposure to air.^5–8^ However, this method fails for other metals, as their higher reactivity with atmospheric oxygen leads to oxide formation that^14,26^ weakens the MoS_2_–metal interaction.^14^ MoS_2_ on different polycrystalline metal films was therefore exfoliated using two controlled environment approaches (details provided in the Supporting Information), which suppress metal oxidation and interfacial contamination.

The first approach utilizes an oxygen- and humidity-free glovebox with an integrated metal deposition system. In addition to Au, we achieved large-area exfoliation of >(100 × 100) μm^2^ MoS_2_ monolayers on Ag, Cu, Pd, Co, and Ni, as shown in Figure 1b–g. The analysis of the optical images reveals that while the overall monolayer yield varies between 45% and 88%, the monolayer selectivity (percentage of monolayer to all MoS_2_ thicknesses) falls within a tight range of 86–99%, which confirms that the monolayer exfoliation selectivity is universal for these metals (Figure S1). The second approach involves exfoliation inside a UHV chamber, attempting to suppress the oxidation and contamination further, which was successful for Au, Cu, Pd, and Ni (Figure S2). Although one may expect UHV-made surfaces to be cleaner than glovebox-made ones, the former often produce smaller monolayers with more cracks than the latter due to lower tactile sensitivity during the remote pressing action inside the UHV chamber. Exfoliation on several other metals was also attempted to no avail (Table S2).

Figure 2a shows how the projected density of electronic states (PDOS) of monolayer MoS_2_ progresses from the most weakly (Au) to the most strongly (Ni) interacting metal. The hybridization of MoS_2_ with the metal gives rise to additional states within the band gap, as detailed in Figure 2b. These states pin the Fermi level (*E*_F_) closer to the conduction band minimum (CBM), indicating n-type behavior.^27^ This deviates from the Schottky–Mott rule, which predicts p-type doping for high-work function metals such as Au, Pd, Co, and Ni.^3,28^ In addition to the changes in *E*_F_, hybridization of the band structure may also alter the electron affinity of MoS_2_ and decrease the work function of monolayer MoS_2_/metal heterostructures below the reported values for MoS_2_ on SiO_2_ (5.15 eV).^29^

**Figure 2:**
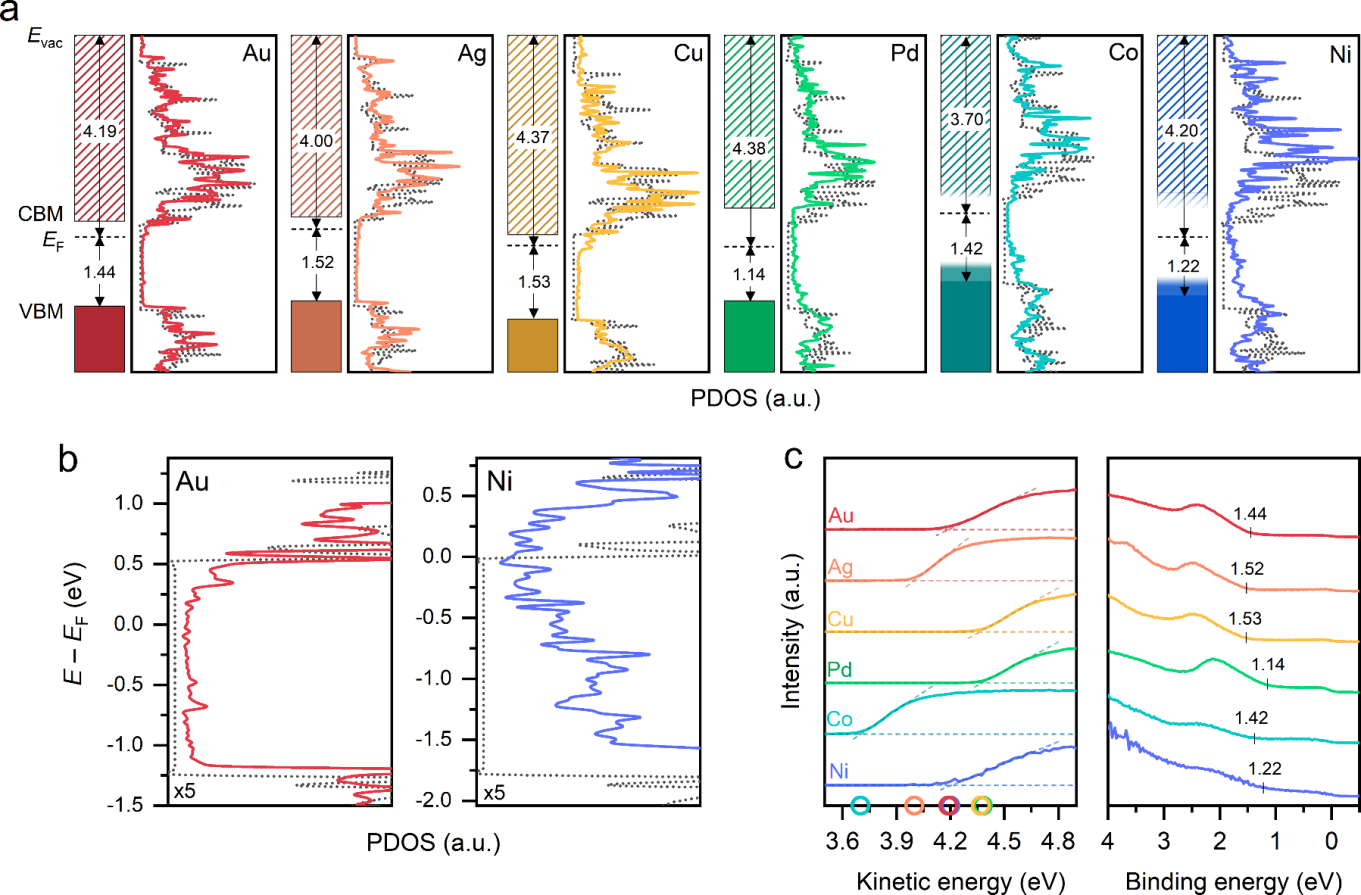
(a) PDOS of monolayer MoS_2_ on Au, Ag, Cu, Pd, Co, and Ni (solid traces) and free-standing MoS_2_ (dotted traces), calculated by DFT. The electronic band structure diagrams on the left-hand side were constructed using the DFT and UPS data. The strong hybridization increases the PDOS within the band gap, which renders the determination of the VBM and CBM less reliable for Pd, Co, and Ni. (b) Magnified midgap state region for MoS_2_ on Au and Ni. (c) UPS spectra of monolayer MoS_2_ on different metals, showing the work function determination from the secondary electron cutoff (left) and VBM determination from the onset of photoemission intensity with respect to *E*_F_ at zero binding energy (right).

Figure 2c shows ultraviolet photoelectron spectroscopy (UPS), which we used to determine the work function and the position of the valence band maximum (VBM) with respect to *E*_F_. We then used these values to construct the band diagrams in Figure 2a (further details in the Supporting Information). Considering the band gap energy of ≈1.8 eV for free-standing MoS_2_ from our DFT calculations or the experimental value of ≈2.1 eV,^30^ the UPS results evidence n-type doping for all of the studied metals.

The role of orbital directionality in the metal-induced effects on the electronic properties of MoS_2_, which may be key to selective monolayer exfoliation, has been overlooked in the literature. To that end, we calculated the *k*-space-resolved electronic states within the backfolded $\sqrt{3}\times \sqrt{3}$ supercell Brillouin zone for the individual Mo and S orbitals. Figure 3 compares the Mo d_*z* ^2^_ and S p_*z*_ out-of-plane orbital contributions for free-standing MoS_2_, MoS_2_ on Au, and MoS_2_ on Ni. The relative blurring of the Mo and S states on Au, and even more so on Ni, and the wispy traces of the metal bands arise from the orbital hybridization between MoS_2_ and the topmost metal layer. Furthermore, there are substantial differences between the top S p_*z*_ (away from the metal) and bottom S p_*z*_ (in contact with the metal) orbital contributions, contrasting those in free-standing MoS_2_. We also observe a relative increase in the mean S–Mo plane distances of the bottom versus top S atoms, which scale with the interaction energy and indicate weakening of the bottom S–Mo bond due to the strong interaction with the metal (Figure S5). These observations could be the underlying reason for the weakening of the vdW interaction between the first and second MoS_2_ layer, which facilitates selective monolayer exfoliation.^4,12,25^ This is because the Mo d_*z* ^2^_ and S p_*z*_ out-of-plane orbital states at the Γ point of the Brillouin zone, which participate in the interlayer MoS_2_–MoS_2_ vdW interaction,^31^ are now disrupted by the strong hybridization with the metal.

**Figure 3:**
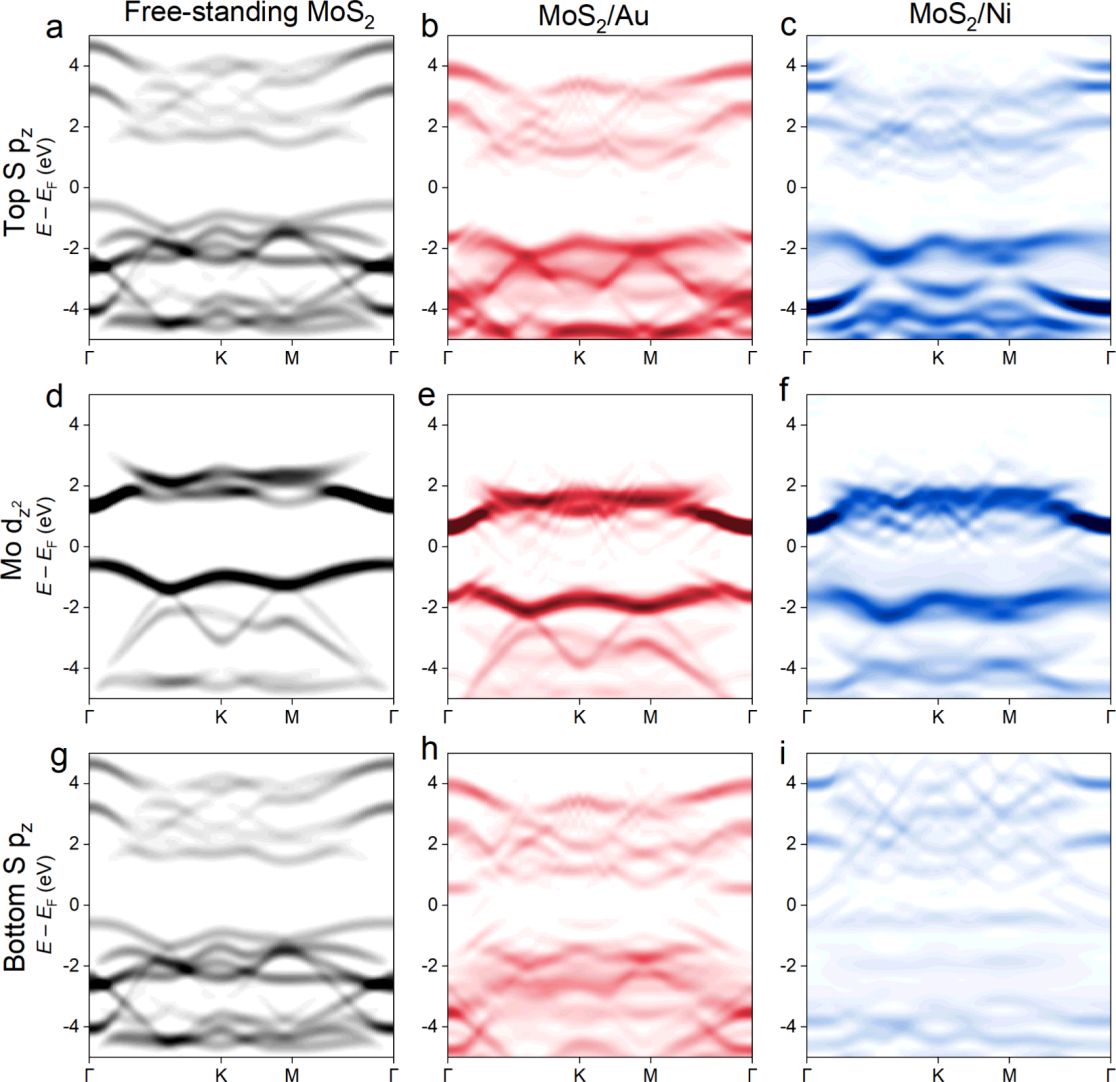
Band structures projected onto the out-of-plane orbitals of free-standing monolayer MoS_2_ (black), monolayer MoS_2_ on Au (red), and monolayer MoS_2_ on Ni (blue) in the backfolded Brillouin zones corresponding to $\sqrt{3}\times \sqrt{3}$ MoS_2_ supercells. (a–c) Top S p_*z*_ orbitals away from the metal, (d–f) Mo d_*z* ^2^_ orbitals, and (g–i) bottom S p_*z*_ orbitals in contact with the metal. In contrast, only small changes were observed in the Mo and S in-plane orbital projections (Figures S3 and S4). Only the spin-up orbital contributions to the band structure of MoS_2_/Ni are shown for the sake of clarity.

The hybridization effects are also observed in the Mo 3d, S 2p, and S 2s core levels measured by X-ray photoelectron spectroscopy (XPS) as shown in Figure 4. Compared with bulk MoS_2_, the core levels of monolayer MoS_2_ on metals are broadened or split and shifted to lower binding energies (*E*_b_). The *E*_b_ downshift cannot be explained solely by a p-type shift of *E*_F_ due to charge transfer, as it partially contradicts the n-type doping observed in the UPS. Instead, the *E*_b_ shifts are likely an aggregate function of both charge transfer and oxidation state changes, reflecting the mixed vdW–covalent nature of the interaction between MoS_2_ and metals and loosely correlating with the propensity of the metal to oxidize. Previous studies of similar effects argued that the downshift of the binding energies originates from the screening of the core levels by the metallic states.^32,33^

**Figure 4:**
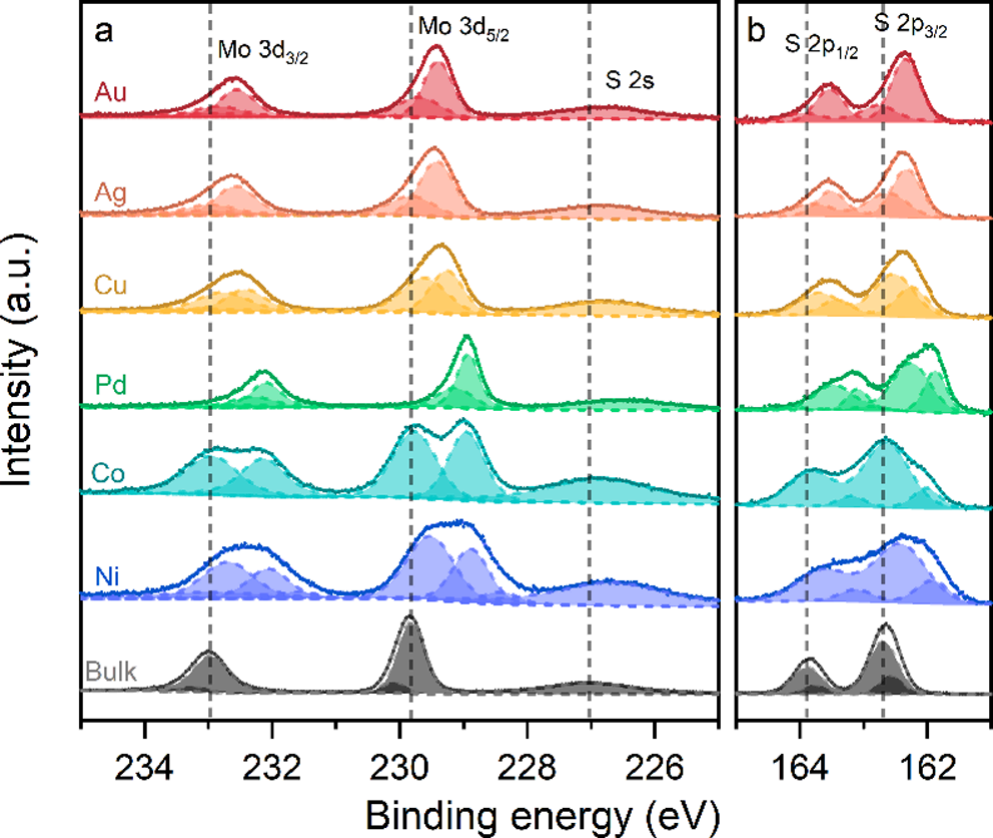
XPS spectra of bulk MoS_2_ (black) and monolayer MoS_2_ exfoliated on Au, Ag, Cu, Pd, Co, and Ni (color). (a) Mo 3d and S 2s core levels and (b) S 2p core levels. Vertical dashed lines indicate the bulk MoS_2_ peak positions as a reference.

Most spectra were fitted with two components, for the Mo 3d and S 2p core levels. The broadening and splitting of the peaks are assigned to the heterogeneous interaction of MoS_2_ with the metals.^11^ The high *E*_b_ component at ≈229.7 eV corresponds to the unperturbed MoS_2_ state, which coincides with the dominant Mo 3d_5/2_ peak of bulk MoS_2_. Conversely, the lower-*E*_b_ component corresponds to the part of MoS_2_, which is in good contact and interacts with the metallic substrate. The downshift of the Mo 3d peak is a consequence of the strong interaction between the underlying metal and the bottom sulfur, which weakens the electron pull of the latter and partially reduces the positive charge of molybdenum.

Several clues imply that the purely covalent bonding of MoS_2_ with the studied metals is unlikely. First, the intrinsic optical and electrical properties of the monolayer MoS_2_ were recovered by oxidizing (Ag)^34^ or etching (Ag and Au) the metal and transferring MoS_2_ to another surface.^35,36^ Second, the typical covalent bond energies, e.g., 3.1 eV per bond in AuS,^37^ exceed the interaction energies even for the most hybridized cases (Pd, Ni, and Co). Therefore, the bond strength should be considered a continuous spectrum of varying interaction energies, rather than two discrete states of a vdW versus covalent bond.^38^ Indeed, weakly hybridized MoS_2_ on Au, Ag, and Cu exhibits the smallest downshifts of the low-*E*_b_ components of Mo 3d and S 2p, while the strongly hybridized Pd, Co, and Ni have larger downshifts.

Furthermore, the splitting of the Mo 3d and S 2p core levels on Co and Ni into two components of similar intensity indicates a substantial amount of noninteracting MoS_2_, arising from the partial oxidation of the less noble Co and Ni surfaces. In contrast, Pd exhibits a lower intensity of the weakly interacting component due to its high nobility as well as a substantial *E*_b_ downshift, consistent with the *E*_F_ downshift observed in UPS. The increasingly metallic character of the Mo atoms on Pd, Co, and Ni due to strong hybridization is also reflected by the higher density of the midgap states and larger deviation from the free-standing MoS_2_ in the PDOS data (Figure 2). The dissimilarity between the XPS spectral weights of the Mo 3d and S 2p states is experimental evidence of the varied chemical environments of the S atoms observed in the PDOS data. Remarkably, MoS_2_ retains its integrity and protects the underlying metal surface from oxidation despite the strong hybridization (Figures S6 and S7).

Changes in Raman spectra of MoS_2_ in contact with Au have been described in our previous studies.^11,14,23^ The interaction between MoS_2_ and Au results in a downshift and broadening of the main E mode (E_2g_^1^ in bulk notation, ≈386 cm^–1^ on SiO_2_) and splitting of the main A_1_ mode (A_1g_ in bulk notation, ≈404 cm^–1^ on SiO_2_). Furthermore, the geometry-forbidden E mode (E_1g_ in bulk notation, ≈279 cm^–1^) and symmetry-forbidden A_1_ mode (A_2u_ in bulk notation, ≈455 cm^–1^) appear. These two modes are inactive in free-standing monolayer MoS_2_ or on noninteracting substrates, such as SiO_2_.^11,23^ The downshift of the E mode was explained by biaxial tensile strain, while the splitting of the A_1_ mode was attributed to n-type doping of MoS_2_ and interaction heterogeneity.^11,38–40^ Weakening of the Mo–S bond was proposed as an alternative explanation for the downshift of the A_1_ mode.^11,41^ Additionally, the general broadening of the peaks in both Raman and X-ray photoelectron spectroscopy observed in this study could be attributed to the polycrystalline nature of the metal films.

Figure 5 summarizes the Raman data of monolayer MoS_2_ exfoliated on Au, Ag, Cu, Pd, Co, Ni, and SiO_2_. Figure 5a–c corresponds to Raman spectra measured with 488 nm excitation, showing the main Raman features. A splitting of the A_1_ mode for Ag and Au, splitting or asymmetry of the E mode for Cu, Pd, and Ni, and general shifts of the modes compared to monolayer MoS_2_ on SiO_2_ are observed. In order to correctly assign the Raman modes, we measured parallel- and cross-polarized Raman spectra of MoS_2_ exfoliated on Au, Ag, and Cu, as shown in Figure 5d–f. The unchanged intensities of one group of the modes and the complete suppression of the other unequivocally confirm the assignment of the E and A_1_ mode symmetries, respectively.^41,42^

**Figure 5:**
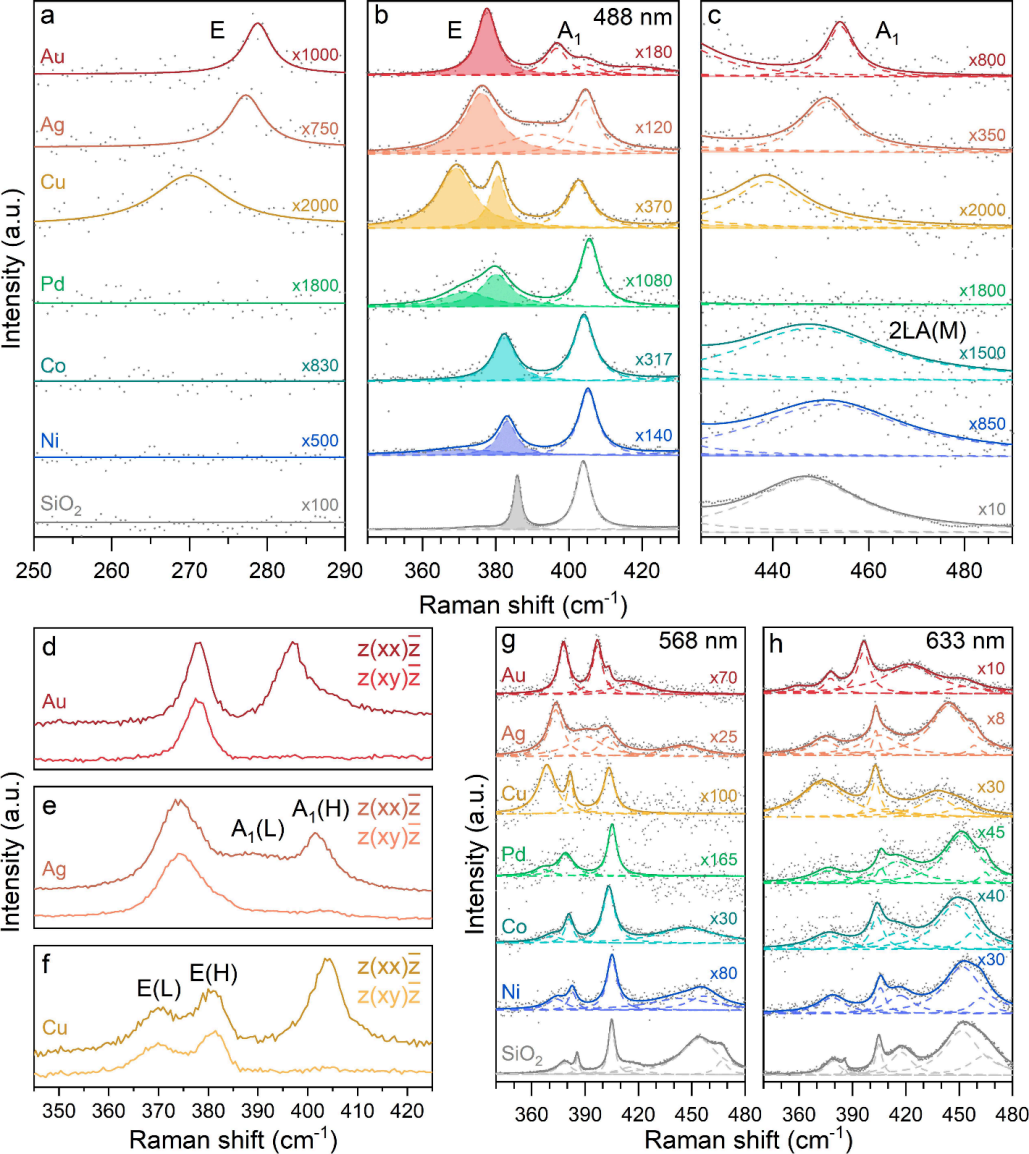
Raman spectra of monolayer MoS_2_ exfoliated on Au, Ag, Cu, Pd, Co, Ni, and SiO_2_ (488 nm excitation): (a) geometry-forbidden E (E_1g_) mode, (b) main E (E_2g_^1^) and A_1_ (A_1g_) modes, and (c) symmetry-forbidden A_1_ (A_2u_) mode and 2LA­(M) Raman mode. Polarized Raman spectra of MoS_2_ on (d) Au, (e) Ag, and (f) Cu for parallel (*z*(*xx*)*z̅*) and perpendicular (*z*(*xy*)*z̅*) polarization of the scattered light with respect to the incident laser (532 nm excitation). (g and h) Raman spectra of monolayer MoS_2_ exfoliated on Au, Ag, Cu, Pd, Co, Ni, and SiO_2_, obtained with 568 and 633 nm excitation, respectively.

The A_1_ (A_1g_) mode splits on Ag into a higher-frequency A_1_(H) mode, which coincides with the A_1g_ mode on SiO_2_, and a broader A_1_(L) mode at a lower frequency. This indicates heterogeneous interaction, most probably due to the higher roughness of the Ag surface than for the other metals and SiO_2_ (Table S3). Our UPS data confirm the n-doping of MoS_2_ on all of the studied metals. However, unlike for n-doping of MoS_2_ on noninteracting SiO_2_,^39^ the A_1_ mode does not downshift or split substantially on Cu, Pd, Co, and Ni. This implies that the effect of n-doping on the A_1_ mode cannot be generalized to strongly interacting substrates and may depend on the additional state occupation in the conduction band. It was shown for n-doped MoS_2_ on Au^43^ and WS_2_ on Au and Ag^32^ that the metal hybridization pulls the conduction band down to *E*_F_, by creating a local minimum at the Q point of the Brillouin zone. This explains the A_1_ mode splitting on Au and Ag, as it was proposed to downshift when the Q and K valleys in the conduction band are simultaneously occupied.^40^

For MoS_2_ on Cu, Pd, and Ni, the E mode splits into high- and low-frequency modes, E­(H) and E­(L), respectively. We rule out the uniaxial strain as a cause since the relative intensities of both E mode peaks remain unchanged during parallel- and cross-polarized measurements (Figure 5f).^44^ Biaxial strain due to a lattice mismatch between MoS_2_ and metal could explain the shift, but the true origin of the splitting remains unclear.

The geometry-forbidden E (E_1g_) and symmetry-forbidden A_1_ (A_2u_) modes are activated on Au, Ag, and Cu (Figure 5a,c), and their shifts correlate with each other and with the main E (E_2g_^1^) mode (Figure 5b). Only the lower-frequency component E­(L) is correlated for Cu. Since the modes with the A symmetry in MoS_2_ are less sensitive to in-plane strain,^45^ this correlation implies that the E modes shift (and split) also for other reasons other than a pure lattice mismatch-induced strain in MoS_2_. The E (E_1g_) and A_1_ (A_2u_) modes are absent for Pd, Co, and Ni and, as expected, for the noninteracting SiO_2_.^23^ The broad feature in the 440–460 cm^–1^ region of the Co and Ni spectra corresponds to the second-order acoustic 2LA­(M) mode also observed for MoS_2_ on SiO_2_.^46,47^ Similar features are also seen with other nonresonant laser wavelengths (Figure S8).

The smaller shifts and lack of significant splitting of the main modes combined with the absence of the forbidden modes on Co and Ni could, at first glance, indicate either a weaker MoS_2_–metal interaction or counterintuitively a very strong interaction. In the latter case, only the phonons from noninteracting MoS_2_ would be observed, whereas the strongly interacting MoS_2_ would acquire a metallic character, thus significantly reducing the Raman scattering cross section.^48^ We exclude the 1H to 1T*′* phase transition of MoS_2_ ^49^ as none of the characteristic 1T*′* modes are observed.^50^

Figure 5g shows the spectra in resonance with the B exciton, with the A_1_ (A_1g_) mode enhanced due to the exciton–phonon coupling.^51^ The higher-order modes also appear on Co and Ni but not on other metals. This strengthens our conclusion that the Raman spectra on Co and Ni correspond to the noninteracting MoS_2_ regions on metal, since a stronger interaction with the substrate suppresses the acoustic modes.^52^ For the 633 nm excitation, we observe the higher-order modes on all samples, probably due to stronger coupling of phonons to the A exciton.^51^ Although the higher-order modes are less intense on Au and Cu, they are stronger on Ag due to its increased roughness, which is evident by the residual photoluminescence signal of the latter. This is in contrast to the complete photoluminescence quenching on all of the other metals due to efficient energy transfer (Figure S9).^11^ The relative changes in the Raman mode intensities at different excitation wavelengths on different metals are also likely influenced by metal-specific plasmon–exciton coupling.^53^ The MoS_2_ mode peak positions for the 488, 514, 532, 568, and 633 nm excitations are listed in Tables S4–S8, respectively.

In summary, our results reveal the effects of polycrystalline metallic substrates on the electronic structure of monolayer MoS_2_ and underscore the crucial role of directional hybridization in the strong interaction between the two materials. The hybridization is likely key to the large-area exfoliation, as it disrupts the out-of-plane orbitals participating in the interlayer vdW interaction in multilayer MoS_2_, weakening the interaction between the first and second MoS_2_ layer. We provide experimental and theoretical evidence for the n-type doping for all studied MoS_2_/metal heterostructures, which is manifested by the Fermi level pinning near the CBM due to the midgap states emerging from hybridization. The wide spectrum of interactions on different metals is evident in Raman spectroscopy and photoemission electron spectroscopy, both of which show associated changes reflecting the interaction strength. Our findings provide consequential insights into the interactions between 2D materials and metals, which has become a rapidly expanding area of research. These insights can be applied to the successful exfoliation of 2D materials on substrates engineered for specific purposes, although they may not fully represent other preparation routes such as the direct epitaxial growth of 2D materials on single crystals.

## Supplementary Material



## Data Availability

The data underlying this study are openly available from the HeyRACK repository at https://doi.org/10.48700/datst.hc3hz-wz038.

## Notes

Details of DFT calculations, sample preparation, and experimental methods and additional results, including figures and tables (PDF)
