# Supplementary material for: Hybridization Directionality Governs the Interaction Strength between MoS2 and Metals
Source: Nano Lett. 2025 Aug 19;25(34):12995–3002. doi: 10.1021/acs.nanolett.5c03200 (PMC12395477; doi:10.1021/acs.nanolett.5c03200)
Supplement: Supplementary file 1 [file nl5c03200_si_001.pdf]

Supporting Information: Hybridization Directionality Governs the Interaction Strength between MoS_2 and Metals

Michaela Hanušová,[†,‡,∇] Luka Pirker,[†,∇] Martin Vondráček,[¶] Václav Valeš,[¶] Casey K. Cheung,[§] Noel Natera Cordero,[§] Amy Carl,[§] Viktor Zólyomi,[‖] János Koltai,[⊥] Ilias Sotiriou,[#] Jens Zscharschuch,[@] Artur Erbe,[@,△] Roman Gorbachev,[§] Jan Honolka,[¶] Otakar Frank,[†] and Matěj Velický[*,†]

†J. Heyrovský Institute of Physical Chemistry, Czech Academy of Sciences (CAS), Dolejškova 2155/3, 182 23 Prague 8, Czech Republic

‡Faculty of Chemical Engineering, University of Chemistry and Technology, Prague, Technická 5, 166 28 Prague 6, Czech Republic

¶Institute of Physics, CAS, Na Slovance 1999/2, 182 21 Prague 8, Czech Republic

§Department of Physics and Astronomy, University of Manchester, Oxford Road, Manchester M13 9PL, United Kingdom

‖Hartree Centre, STFC Daresbury Laboratory, Daresbury WA4 4AD, United Kingdom

⊥Department of Biological Physics, Eötvös Loránd University, Pázmány Péter sétány 1/A, Budapest 1117, Hungary

#Institute of Electronic Packaging Technology, Technische Universität Dresden, 01062 Dresden, Germany

@Institute of Ion Beam Physics and Materials Research, Helmholtz-Zentrum Dresden-Rossendorf, Bautzner Landstrasse 400, 01328 Dresden, Germany

△Technische Universität Dresden, 01069 Dresden, Germany

∇Contributed equally to this work

E-mail: matej.velicky@jh-inst.cas.cz

Table of contents

S1 Density Functional Theory Calculations **3**

S2 Sample Preparation **4**

S2.1 Glovebox Exfoliation 4

S2.2 UHV Exfoliation 4

S3 Methods **5**

S3.1 Photoemission Spectroscopy 5

S3.2 Optical Spectroscopy and Microscopy 6

S3.3 Atomic Force Microscopy 6

S4 Additional Results **7**

S1 Density Functional Theory Calculations

We used the Quantum Espresso density functional theory (DFT) code[1,2] to compute the optimal structure, interaction energy, and projected density of states (PDOS) of monolayer (1L) MoS_2 deposited on the close-packed surfaces of the metal slabs. We used the (111) surface for fcc crystals and the (0001) surface for hcp crystals. Slab thickness was set to 8 layers and the vacuum regime to a width of 12 Å, which was found sufficient to converge the interaction energy to a meV/Å^2 precision. Full surface relaxation is performed on the freestanding metal slabs, starting from a structure determined by the DFT lattice constant in the bulk crystal. During the structural optimization of the superstructure, the bottom half of the layers is kept fixed. We used the PBEsol density functional and a plane-wave cutoff energy of 680.3 eV (50 Ry). The k-point density for the 1L MoS_2 was set to $12 \times 12 \times 1$ and this was proportionally reduced in the superstructures corresponding to the supercell size. Pseudopotentials for the metal slabs were taken from the SSSP Precision library[3] for the metal slabs, while for the MoS_2 we chose the pslibrary 1.0.0 pseudopotentials.[4] This is because the pslibrary pseudopotentials performed better in tests for the critically important lattice parameter of MoS_2, but they failed to reproduce the known surface relaxation on Au fcc (111), in which the SSSP pseudopotentials succeeded. Given that SSSP already mixes different flavors or pseudopotentials, we anticipate no issues from using pslibrary for MoS_2 in conjunction with it. When constructing the supercells, we applied a compensating strain to the metal slabs to achieve commensurability, as required by the periodic boundary conditions, as shown in Supporting Table S1. For Ni and Co, spin-polarization was taken into account.

S2 Sample Preparation

S2.1 Glovebox Exfoliation

290 nm SiO_2/Si wafers were sonicated first in acetone and then in isopropyl alcohol, followed by 1-min cleaning using either Ar/O_2 or pure O_2 plasma. Polycrystalline metal films were deposited onto the cleaned wafers using a custom-made e-beam evaporator sytem (MBraun) with SmartBeam controller and Inficon deposition monitor or EB-6 e-beam evaporator (BeamTec) with the SQC-310 Inficon controller. 3 nm Cr or 3 nm Ti were used as adhesion layers. The thickness of the metallic substrate (Ag, Al, Au, Bi, Cu, Co, Cr, Fe, In, Ni, Pd, Pt, Ti) was 15 nm (10 nm of the Co substrate). Bulk MoS_2 (Manchester Nanomaterials Ltd) was cleaved and directly exfoliated onto the freshly deposited metallic substrate using the scotch-tape technique inside an Ar atmosphere of the MB200MOD glovebox (MBraun) with oxygen content kept under 2 ppm and water levels between 2-6 ppm.

S2.2 UHV Exfoliation

Two UHV exfoliation procedures were used. The first one involved metal deposition and direct exfoliation inside the UHV multi-chamber cluster tool commissioned by PREVAC. The cleaned SiO_2/Si wafers were clamped onto PTS sample holders (PREVAC) and loaded into the UHV chamber, followed by O_2 plasma cleaning. The substrate metal (15 nm) was then deposited via e-beam evaporation (Ag, Al, Au, Cu, Pd, Sn, Ti) or magnetron sputtering (Pt, Ta), on top of a 3 nm Ti adhesion layer. The bulk MoS_2 crystal stamp was cleaved inside the UHV tool also, brought into contact with the metallic substrate, and pressed against it with a force of 5 N for 5 minutes.

The second procedure involved the deposition of a 3 nm Cr adhesion layer and 20 nm metallic substrate (Ag, Au, Cu, Co, Ni) via magnetron sputtering (Q300TD, Quorum Technologies). The substrates were briefly exposed to air and then loaded into the UHV

chamber, where Ar sputtering was used to remove the surface oxides and other contaminants. The oxidation and adventitious carbon content of the metallic surface were assessed in situ by XPS. The bulk MoS_2 stamp was cleaved under the UHV and pressed down onto the metallic substrate using a custom-made manipulator.

S3 Methods

S3.1 Photoemission Spectroscopy

Ultraviolet photoelectron spectroscopy (UPS) and X-ray photoelectron spectroscopy (XPS) were measured using a NanoESCA instrument (Omicron) with He I ($h\nu$ = 21.2 eV) and monochromatized Al Kα ($h\nu$ = 1486.7 eV) light sources, respectively. The photoemission setup includes an energy-filtered photoemission electron microscopy (PEEM) mode. Through the work function contrast, PEEM imaging can identify exposed metal areas and differentiate between monolayer and multilayer areas of MoS_2.

The Al Kα focused X-ray beam has a spot size of about $\approx (60 \times 100)$ µm^2 on the sample surface. The selected areas of 1L MoS_2 on the six metals described in the manuscript were larger than the spot size. Apart from the work function contrast from PEEM, the MoS_2 thickness is also confirmed by the characteristic intensity ratio between the metal and Mo core levels. The ratio between Au $4f_{7/2}$ and Mo 3d core level intensities is approximately 6 for monolayer MoS_2 on gold, which decreases to 2 for the bilayer due to the increased attenuation of the Au signal via inelastic electron scattering processes in the presence of an additional MoS_2 layer. In the case of UPS, the analyzed area of $\approx (50 \times 50)$ µm^2 is defined by the electron optics after the photoemission process. For both XPS and UPS, the analyzed area can be further reduced to $\approx (20 \times 20)$ µm^2 by closing the iris aperture, when required.

XPS core level spectra were fitted with Voigt peaks on a Shirley background using the KolXPD software. The energy scale was calibrated using the Fermi edge and Au $4f_{7/2}$ peak. Surface charging was suppressed by grounding the metallic substrate during measurements.

The UPS data shown in the left panel of Fig. 2c on the kinetic energy scale (main text) is calibrated in such a way that the electrons emitted from the Fermi level appear at a kinetic energy equal to the incident photon energy of 21.2 eV. The work function is then equal to the kinetic energy at the secondary electron cut-off. Valence band maxima (VBM) were determined from the onset of the UPS curve on the binding energy scale, as shown in the right panel of Fig. 2c (main text). Here, the Fermi level is positioned at $E_\mathrm{b} = 0$ eV. The VBM and conduction band minimum (CBM) positions in the band structure diagrams of Fig. 2a in the main text were aligned according to their calculated PDOS intensity onsets.

S3.2 Optical Spectroscopy and Microscopy

The unpolarized Raman and photoluminescence (PL) spectra were measured using the LabRAM HR spectrometer (Horiba Jobin-Yvon) with 488, 514, 568, and 633 nm excitation laser focused through 100x objective (Olympus, MPlan N), and 1800 l/mm grating for the high-resolution Raman spectra and 600 l/mm grating for the PL spectra. The Raman spectra obtained with 532 nm excitation laser, focused through 100x objective (Zeiss EC Epiplan-Neofluar), were measured using a 1800 l/mm grating on a WITec Alpha 300 R (Oxford Instruments).

The polarized Raman spectra were obtained using the LabRAM HR Evolution spectrometer with the 532 nm excitation wavelength, 1800 l/mm grating, and a 100x objective (Olympus, MPlan N). In all measurements, the laser power was maintained below 0.5 mW to prevent damage to the samples.

S3.3 Atomic Force Microscopy

Atomic force microscopy (AFM) images were acquired using the Cypher-S AFM (Oxford Instruments) in an argon-atmosphere glovebox. The ambient-environment AFM was measured using the Dimension Icon (Bruker Nano) in PeakForce QNM mode and the OmegaScope-R SPM/Optical system (HORIBA Scientific) in AC mode.

S4 Additional Results

Table S1: Compensating strain for the different sizes of the MoS_2/metal supercell (in lattice parameter multiples), applied to achieve commensurability between the two materials.

metal	MoS_2 size	metal slab size	strain (%)
Au	$\sqrt{3} \times \sqrt{3}$	2×2	-5.7
Au	$\sqrt{7} \times \sqrt{7}$	3×3	1
Ag	$\sqrt{3} \times \sqrt{3}$	2×2	-5.2
Ag	$\sqrt{7} \times \sqrt{7}$	3×3	1
Cu	$\sqrt{3} \times \sqrt{3}$	2×2	7.8
Cu	$\sqrt{7} \times \sqrt{7}$	$\sqrt{12} \times \sqrt{12}$	-4.9
Pd	$\sqrt{3} \times \sqrt{3}$	2×2	-0.8
Pd	$\sqrt{7} \times \sqrt{7}$	3×3	1
Co	$\sqrt{3} \times \sqrt{3}$	2×2	12.4
Co	2×2	$\sqrt{7} \times \sqrt{7}$	1.88
Ni	$\sqrt{3} \times \sqrt{3}$	2×2	11.2
Ni	2×2	$\sqrt{7} \times \sqrt{7}$	-2.9

Table S2: Record of further unsuccessful exfoliation attempts on a range of different metals. Exfoliation in a glovebox yielded bulk MoS_2 on In or negligible amounts of any MoS_2 thickness on Cr, Fe, Pt, and Bi. Exfoliation under UHV was unsuccessful on Al, Ti, Ag, Sn, Co, and Pt, due to a variety of contributing factors.

<table>
<tr><th>environment</th><th>metal</th><th>exfoliation yield</th></tr>
<tr><td rowspan="5">glovebox</td><td>In</td><td>only bulk</td></tr>
<tr><td>Cr</td><td rowspan="4">negligible</td></tr>
<tr><td>Fe</td></tr>
<tr><td>Pt</td></tr>
<tr><td>Bi</td></tr>
<tr><td rowspan="6">UHV</td><td>Al</td><td rowspan="6">negligible</td></tr>
<tr><td>Ti</td></tr>
<tr><td>Ag</td></tr>
<tr><td>Sn</td></tr>
<tr><td>Co</td></tr>
<tr><td>Pt</td></tr>
</table>

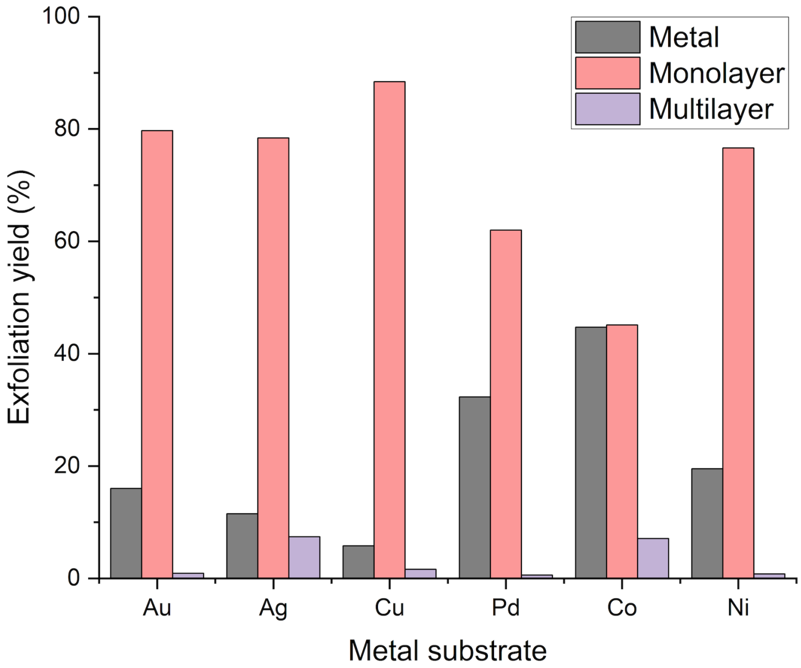

Figure S1: MoS_2 exfoliation yield as a percentage of the total area of the optical image taken with a 10x objective for different metallic substrates, confirming that the monolayer exfoliation selectivity is universal for these metals. The overall monolayer yield ranges from 45% to 88%, while the monolayer selectivity varies from 86% to 99%.

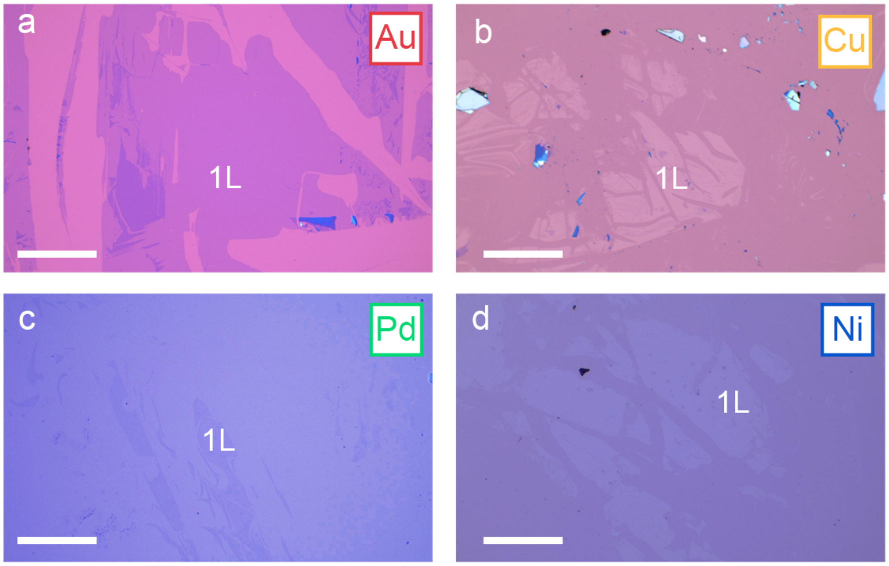

Figure S2: Optical images of 1L MoS_2 exfoliated on Au, Cu, Pd, and Ni under UHV. The scale bars correspond to 200 µm.

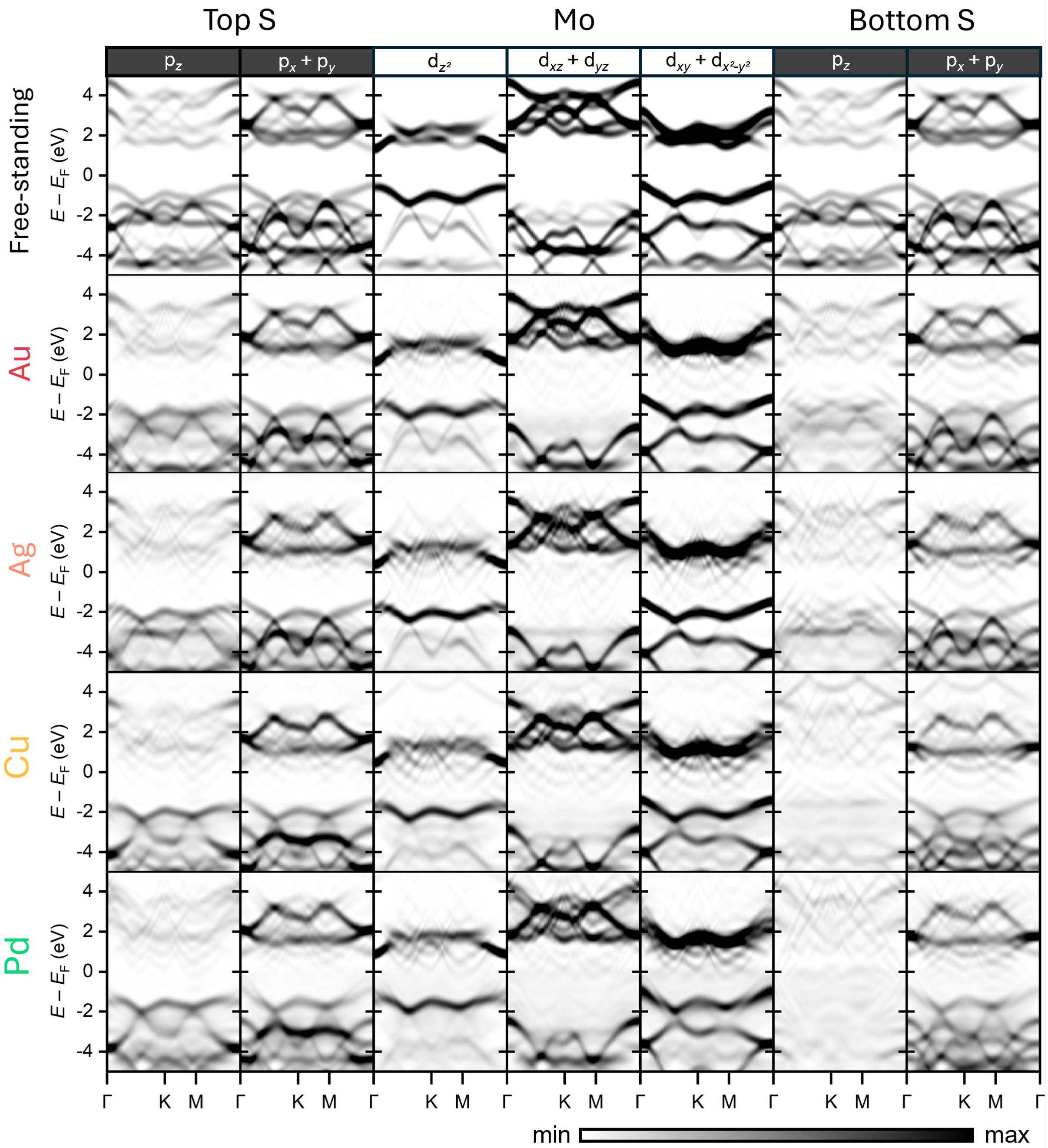

Figure S3: Band structures projected onto the in-plane ($p_x + p_y$, $d_{xy} + d_{x^2-y^2}$) and out-of-plane (p_z, d_{z^2}, $d_{xz} + d_{yz}$) orbitals of freestanding 1L MoS_2 (black) and 1L MoS_2 on Au (red), Ag (orange), Cu (yellow), and Pd (green) in the backfolded Brillouin zones corresponding to $\sqrt{3} \times \sqrt{3}$ MoS_2 supercells. The out-of-plane orbital projections show progressively larger changes going from the weakly (Au) to the moderately (Pd) interacting metal, while little changes were observed for the in-plane orbital projections.

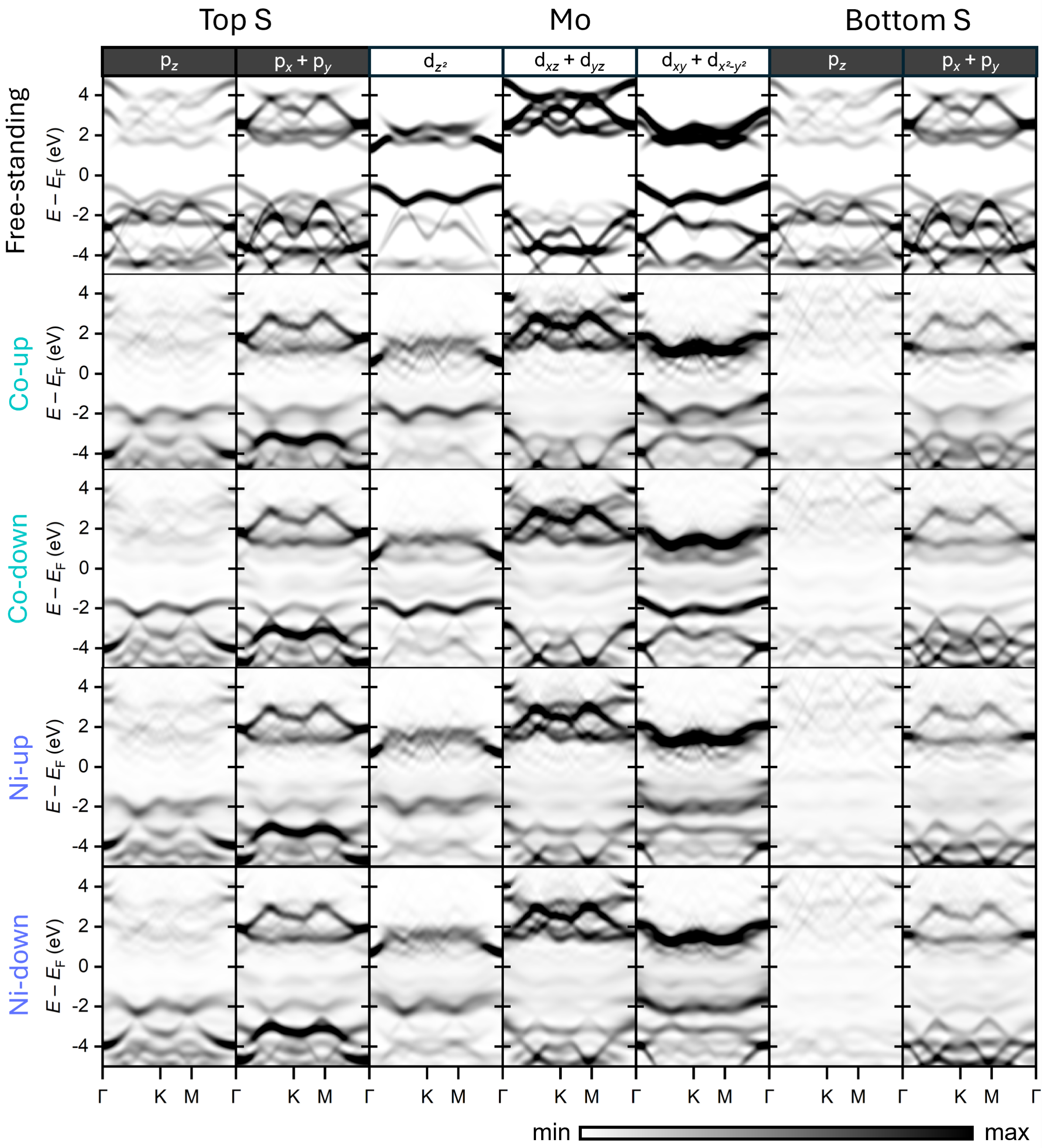

Figure S4: Band structures projected onto the in-plane and out-of-plane orbitals of free-standing 1L MoS_2 (black) and 1L MoS_2 on Co (turquoise) and Ni (blue) in the backfolded Brillouin zones corresponding to $\sqrt{3} \times \sqrt{3}$ MoS_2 supercells. Both spin-up and spin-down contributions are shown. The out-of-plane orbital projections are significantly affected by the hybridization for both metals, while only moderate changes are observed for the in-plane orbital projections.

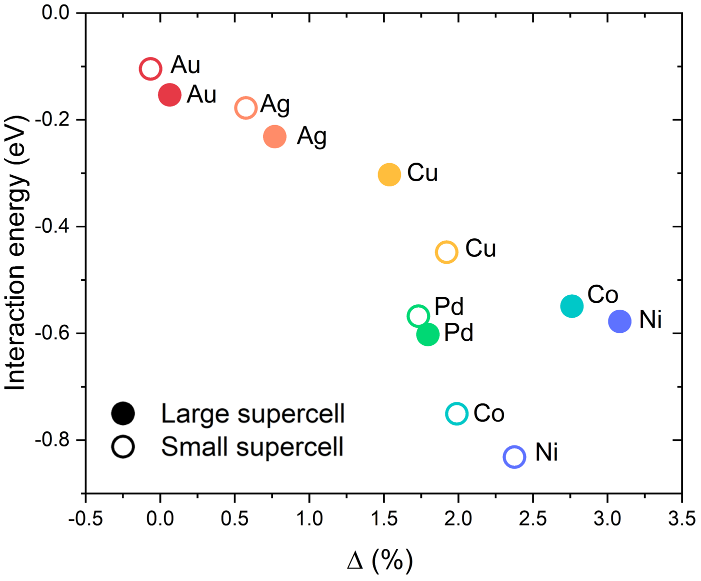

Figure S5: Interaction energy correlation with the relative difference between the mean S-Mo plane distances of the bottom ($d_{\text{Mo-S}_{\text{bottom}}}$) and top ($d_{\text{Mo-S}_{\text{top}}}$) S atoms in the 1L MoS_2/metal heterostructures, calculated by the DFT using the small and large supercells and expressed as $\Delta = 100 \times (d_{\text{Mo-S}_{\text{bottom}}} - d_{\text{Mo-S}_{\text{top}}})/d_{\text{Mo-S}_{\text{top}}}$ (%).

Table S3: Root mean square (RMS) roughness of the metallic surfaces measured by a Cypher-S AFM before their exposure to air. Ag has a notably higher RMS roughness than the other metals and SiO_2 (measured by the Dimension Icon AFM).

sample	deposition rate (Å/s)	RMS (Å)
SiO_2	–	2.1
Ag	1.0	7.4
Cu	1.0	4.2
Pd	0.3	2.6

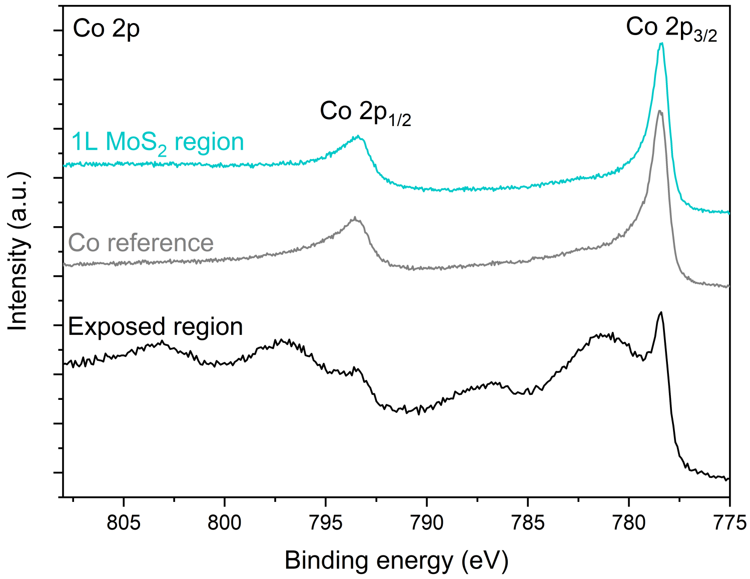

Figure S6: XPS spectra of the Co 2p core levels from the region covered with 1L MoS_2 (turquoise), reference Co sample cleaned *in situ* under the UHV (grey), and exposed metal region without MoS_2 (black). The sharp asymmetric peaks (1L MoS_2 region and Co reference) correspond to metallic Co, while the broader peaks at higher E_b (exposed region) correspond to cobalt oxide core levels and shake-up satellites. This data shows that 1L MoS_2 protects the underlying metal from oxidation, which is also why the 1L MoS_2 appears to be the same or lower height than the bare metal surrounding it (Supporting Fig. S7).

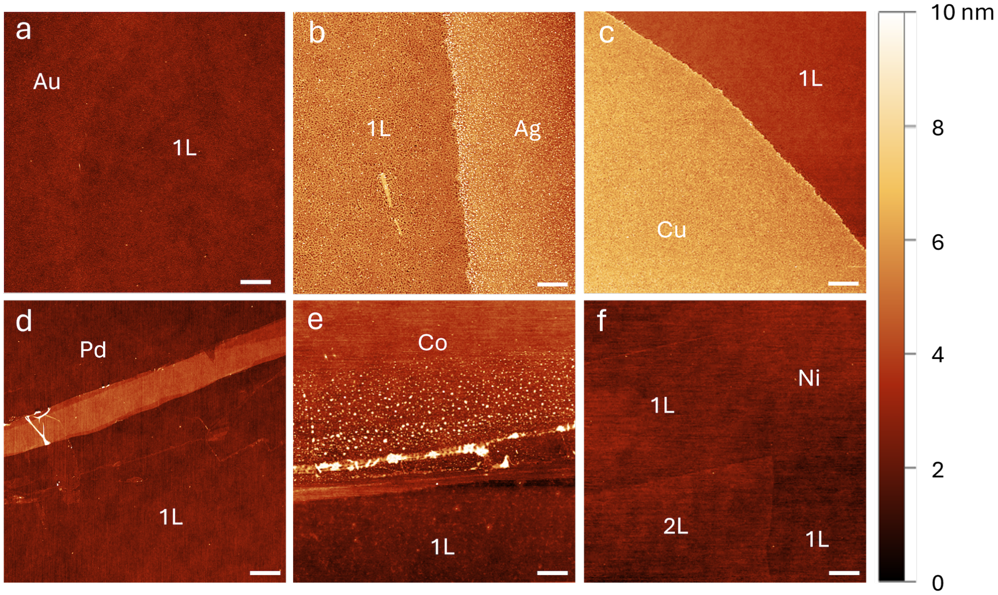

Figure S7: AFM images of 1L MoS_2 exfoliated on metals in a glovebox. The MoS_2 *versus* bare metal height inconsistencies indicate oxidation of the exposed metal. The scale bar corresponds to 1 µm.

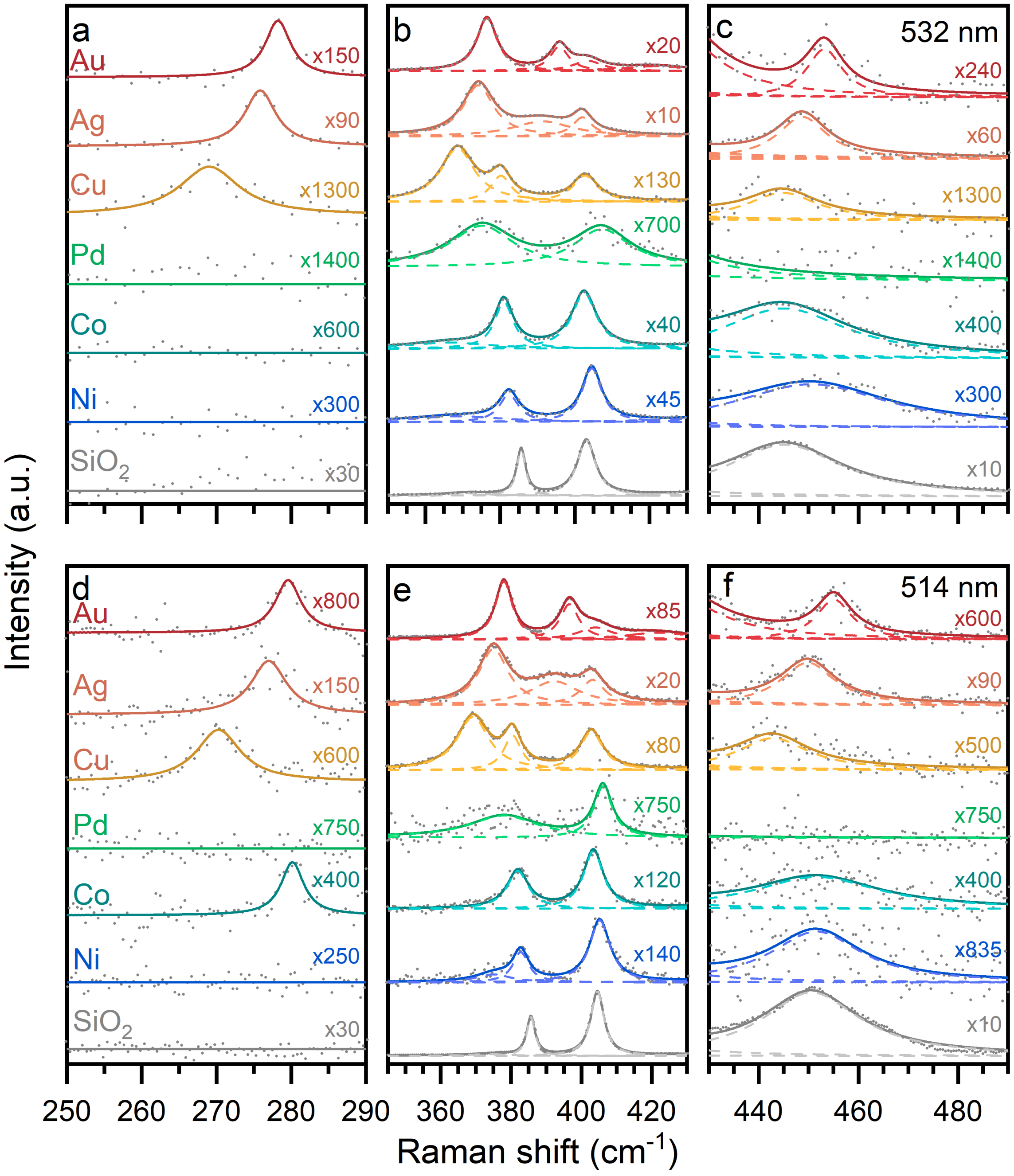

Figure S8: Raman spectra of 1L MoS_2 exfoliated on Au, Ag, Cu, Pd, Co, Ni, and SiO_2 obtained with 532 nm (a–c) and 514 nm (d–f) excitation wavelength.

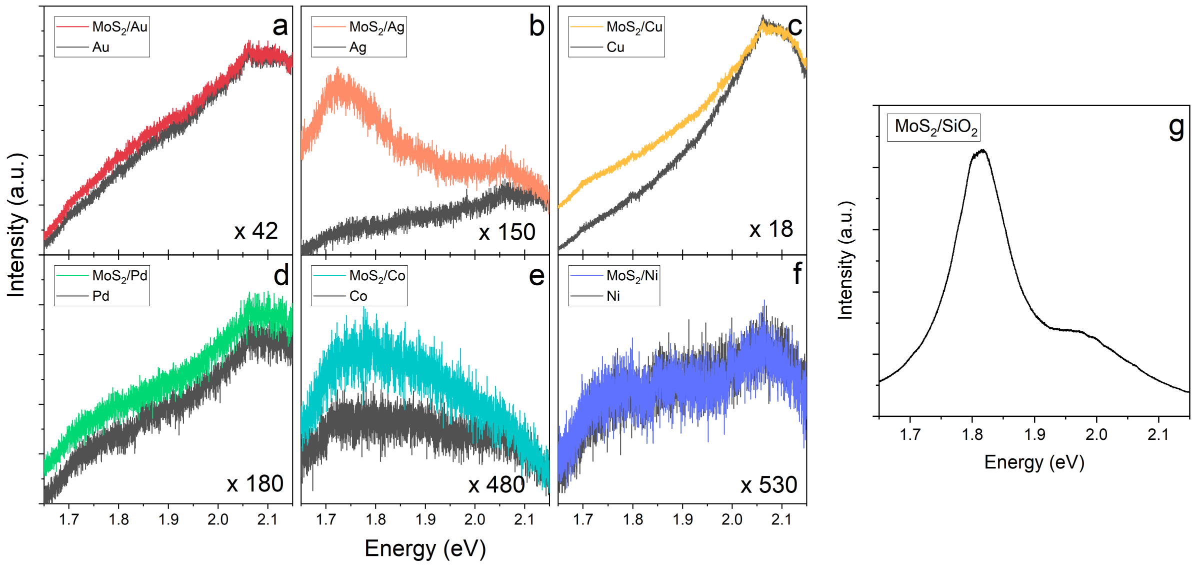

Figure S9: PL spectra of 1L MoS_2 on (a) Au, (b) Ag, (c) Cu, (d) Pd, (e) Co, (f) Ni compared to spectra of bare metals, obtained with 514 nm excitation wavelength. (g) PL spectrum of 1L MoS_2 exfoliated on SiO_2.

Table S4: Fitted 1L MoS_2 peak positions (cm^{-1}) on different metals (488 nm excitation). The modes are labeled using notation for 1L MoS_2 with a broken symmetry due to the interaction with the substrate.[5]

sample	E	E(L)	E(H)	A_1(L)	A_1(H)	LA(K)+TA(K)	A_1/2LA(M)
Au	278.8	–	377.6	396.7	405.0	419.8	454.0
Ag	277.3	–	376.0	391.5	404.6	–	451.1
Cu	269.9	369.2	380.5	–	402.7	–	438.8
Pd	–	371.8	380.1	–	405.6	–	–
Co	–	–	382.3	–	404.1	–	448.0
Ni	–	371.2	383.1	–	405.2	–	451.6
SiO_2	–	374.2	385.9	–	403.9	–	446.9

Table S5: Fitted 1L MoS_2 peak positions (cm^{-1}) on different metals (514 nm excitation).

sample	E	E(L)	E(H)	A_1(L)	A_1(H)	LA(K)+TA(K)	A_1/2LA/(M)
Au	279.6	–	378.0	396.6	404.0	421.8	455.1
Ag	277.0	–	375.0	392.2	403.2	–	449.8
Cu	270.3	369.2	380.4	–	402.9	–	442.8
Pd	–	–	378.0	–	406.1	–	–
Co	280.2	–	381.9	–	403.4	–	451.6
Ni	–	375.0	382.7	–	405.2	–	451.5
SiO_2	–	374.3	385.7	–	404.5	–	452.0

Table S6: Fitted 1L MoS_2 peak positions (cm^{-1}) on different metals (532 nm excitation).

sample	E	E(L)	E(H)	A_1(L)	A_1(H)	LA(K)+TA(K)	A_1/2LA(M)
Au	278.2	–	376.5	395.9	403.5	421.5	453.2
Ag	275.9	336.5	374.2	391.4	402.1	–	448.7
Cu	269.1	368.6	380.2	–	402.7	–	444.8
Pd	–	–	375.2	–	407.2	–	–
Co	–	366.5	381.0	–	402.5	–	445.0
Ni	–	368.6	382.4	–	404.6	–	450.5
SiO_2	–	371.2	385.6	–	403.1	–	445.2

Table S7: Fitted 1L MoS_2 peak positions (cm^{-1}) on different metals (568 nm excitation).

sample	LO(M)	E(L)	E(H)	A_1(L)	A_1(H)	LA(K)+TA(K)	A_1/2LA(M)
Au	–	–	377.9	396.8	403.3	415.6	–
Ag	–	–	373.5	390.4	402.7	–	445.6
Cu	–	368.5	381.6	–	403.4	–	–
Pd	–	366.3	379.1	–	405.2	–	–
Co	–	371.8	380.9	–	403.3	–	447.4
Ni	–	374.0	382.8	–	405.1	–	443.8 – 456.7
SiO_2	378.3	–	385.5	–	404.9	415.1	454.1 – 466.9

Table S8: Fitted 1L MoS_2 peak positions (cm^{-1}) on different metals (633 nm excitation).

sample	LO(M)	E(L)	E(H)	A_1(L)	A_1(H)	LA(K)+TA(K)	A_1/2LA(M)
Au	359.0	–	377.6	–	396.2	422.1	452.9
Ag	–	–	374.3	403.1	405.4	–	444.1 – 458.4
Cu	–	–	373.3	–	402.8	410.1	437.3 – 450.5
Pd	–	–	376.3	–	405.9	415.0	451.2 – 464.5
Co	–	–	376.3	–	403.7	415.0	447.6 – 457.5
Ni	–	–	378.6	–	405.6	416.7	451.8 – 462.2
SiO_2	378.9	–	385.4	–	404.7	417.3	451.4 – 464.2

Table S9: XPS fiting parameters. Δ Mo is the difference between Mo $3d_{5/2}$(H) and Mo $3d_{5/2}$(L), Δ S is the difference between S $2p_{3/2}$(H) and S $2p_{3/2}$(L). Additional peaks: for Ni 231.60 eV and 228.42 eV; for SiO_2 232.33 eV and 229.19 eV.

sample	Mo $3d_{3/2}$(H)	Mo $3d_{3/2}$(L)	Mo $3d_{5/2}$(H)	Mo $3d_{5/2}$(L)	S 2s	Δ Mo
Au	232.85	232.55	229.70	229.40	226.80	0.30
Ag	232.97	232.57	229.82	229.42	226.81	0.40
Cu	232.78	232.42	229.62	229.26	226.79	0.36
Pd	232.39	232.15	229.22	228.97	226.48	0.25
Co	232.95	232.14	229.77	228.95	226.85	0.82
Ni	232.70	232.06	229.52	228.89	226.67	0.64
bulk	233.22	232.97	230.08	229.82	227.02	0.26

sample	S $2p_{1/2}$(H)	S $2p_{1/2}$(L)	S $2p_{3/2}$(H)	S $2p_{3/2}$(L)	Δ S
Au	163.95	163.53	162.76	162.35	0.42
Ag	163.81	163.51	162.63	162.33	0.30
Cu	163.71	163.42	162.53	162.24	0.28
Pd	163.47	163.11	162.28	161.92	0.37
Co	163.82	163.19	162.64	162.01	0.63
Ni	163.63	163.12	162.43	161.92	0.51
bulk	163.89	163.76	162.69	162.57	0.12

References

1. Giannozzi, P.; Baroni, S.; Bonini, N.; Calandra, M.; Car, R.; Cavazzoni, C.; Ceresoli, D.; Chiarotti, G. L.; Cococcioni, M.; Dabo, I. *et al.* QUANTUM ESPRESSO: A Modular and Open-source Software Project for Quantum Simulations of Materials. *Journal of Physics: Condensed Matter* **2009**, *21*, 395502.

2. Giannozzi, P.; Andreussi, O.; Brumme, T.; Bunau, O.; Nardelli, M. B.; Calandra, M.; Car, R.; Cavazzoni, C.; Ceresoli, D.; Cococcioni, M. *et al.* Advanced Capabilities for Materials Modelling with QUANTUM ESPRESSO. *Journal of Physics: Condensed Matter* **2017**, *29*, 465901.

3. Prandini, G.; Marrazzo, A.; Castelli, I. E.; Mounet, N.; Marzari, N. Precision and Efficiency in Solid-State Pseudopotential Calculations. *npj Computational Materials* **2018**, *4*, 72.

4. Dal Corso, A. Pseudopotentials Periodic Table: From H to Pu. *Computational Materials Science* **2014**, *95*, 337–350.

5. Rodríguez, A.; Velický, M.; Řáhová, J.; Zólyomi, V.; Koltai, J.; Kalbáč, M.; Frank, O. Activation of Raman Modes in Monolayer Transition Metal Dichalcogenides Through Strong Interaction With Gold. *Physical Review B* **2022**, *105*, 195413.
